# Tumor Tissue Explant Culture of Patient-Derived Xenograft as Potential Prioritization Tool for Targeted Therapy

**DOI:** 10.3389/fonc.2019.00017

**Published:** 2019-01-22

**Authors:** Susmita Ghosh, Manu Prasad, Kiran Kundu, Limor Cohen, Ksenia M. Yegodayev, Jonathan Zorea, Ben-Zion Joshua, Batel Lasry, Orr Dimitstein, Anat Bahat-Dinur, Aviram Mizrachi, Vladimir Lazar, Moshe Elkabets, Angel Porgador

**Affiliations:** ^1^The Shraga Segal Department of Microbiology, Immunology and Genetics, Faculty of Health Sciences, Ben-Gurion University of the Negev, Beer Sheva, Israel; ^2^National Institute for Biotechnology in the Negev, Ben-Gurion University of the Negev, Beer Sheva, Israel; ^3^Department of Otolaryngology-Head and Neck Surgery, Soroka Medical Center and Faculty of Health Sciences, Ben-Gurion University of the Negev, Beer Sheva, Israel; ^4^Department of Otolaryngology-Head and Neck Surgery and The Center for Translational Research in Head and Neck Cancer, Rabin Medical Center, Petah Tikva and Sackler Faculty of Medicine, Tel Aviv University, Tel Aviv, Israel; ^5^Worldwide Innovative Network Association-WIN Consortium, Villejuif, France

**Keywords:** head and neck cancer, patient derived xenografts, *ex vivo*, explant culture, targeted therapy

## Abstract

Despite of remarkable progress made in the head and neck cancer (HNC) therapy, the survival rate of this metastatic disease remain low. Tailoring the appropriate therapy to patients is a major challenge and highlights the unmet need to have a good preclinical model that will predict clinical response. Hence, we developed an accurate and time efficient drug screening method of tumor *ex vivo* analysis (TEVA) system, which can predict patient-specific drug responses. In this study, we generated six patient derived xenografts (PDXs) which were utilized for TEVA. Briefly, PDXs were cut into 2 × 2 × 2 mm^3^ explants and treated with clinically relevant drugs for 24 h. Tumor cell proliferation and death were evaluated by immunohistochemistry and TEVA score was calculated. *Ex vivo* and *in vivo* drug efficacy studies were performed on four PDXs and three drugs side-by-side to explore correlation between TEVA and PDX treatment *in vivo*. Efficacy of drug combinations was also ventured. Optimization of the culture timings dictated 24 h to be the time frame to detect drug responses and drug penetrates 2 × 2 × 2 mm^3^ explants as signaling pathways were significantly altered. Tumor responses to drugs in TEVA, significantly corresponds with the drug efficacy in mice. Overall, this low cost, robust, relatively simple and efficient 3D tissue-based method, employing material from one PDX, can bypass the necessity of drug validation in immune-incompetent PDX-bearing mice. Our data provides a potential rationale for utilizing TEVA to predict tumor response to targeted and chemo therapies when multiple targets are proposed.

## Introduction

Advances in basic and medical science research led to the discovery of numerous cancer specific-targeted chemotherapy drugs [reviewed in (1)]. The variety of treatment alternatives and the existence of several mutated signaling pathways in each cancer imposes a new challenge to the physicians since omics-based prediction of personalized treatment might not suffice to accurately predict and prioritize the best personalized treatment [reviewed in (2)]. Therefore, additional test of the various omics-predicted targeted therapies, on the specific patient's cancer, is needed. At present, several *in vitro* 3D test systems, based on human material taken from patient's cancer biopsy or surgery, are investigated for the evaluation of optimal personalized targeted chemotherapy regimen [reviewed in (3, 4)]. Alternatively, growing patient derived xenografts (PDXs) and evaluating response to targeted chemotherapies *in vivo* is also studied [reviewed in (5)]. Both methods have their limitations; particularly the shortage in human material taken from biopsy/surgery of small lesions (for the *in vitro* 3D approach) and length of time needed to obtain adequate number of PDX's to evaluate *in vivo* several targeted drug candidates. Therefore, there is an unmet need of a proper preclinical system that can be employed for assessing the optimal targeted single drug or combination from a list of omics-predicted targeted drugs. In addition, for the scenario that patient's tumor omics do not result in list of available targeted drugs, there is a need to assess the effect of quite a few off-label drugs on the patient's tumor to try to come with a possible candidate drug.

Head and neck cancer (HNC) is the sixth most common cancer worldwide where only 40–50% of the patients have a survival rate of nearly 5-years (6). For early stage disease, surgery and/or radiotherapy are the only standard of care (7). However, for locoregionally advanced stage disease, cisplatin-based chemo-radiotherapy remains the first treatment of choice while cetuximab is for platinum-based chemotherapy resistant patients (8, 9). For recurrent and metastatic disease, addition of cetuximab to platinum-based chemotherapy offers modest survival benefit (10). Cetuximab is the only FDA approved targeted monoclonal antibody against epidermal growth factor receptor (EGFR) for HNC patients. When administered along with platinum-based chemotherapy, it has no correlation to either EGFR copy number or level of EGFR expression in predicting its response (11, 12). Presently, in the myriad of treatment options, no universally agreed second line therapy exists.

In such a scenario, prediction of drug responses by employing patient derived xenograft (PDX) models has been done by many researchers, imparting ingenious advantages as a preclinical model (5, 13–17). Yet, as aforementioned, exploring drug efficiency in mice is costly as well as time taking in clinical decision making. Hence, oncology research with PDX model can be regarded as more suitable in drug validation upon drug screening (18). As discussed, extensive research efforts have been seen to develop *ex vivo* drug efficacy assays. Such efforts include isolation of fresh tumor cells from patients (19), patient derived 3D tumor spheroids (20, 21), or 3D organoids (22, 23), tumor tissue slices (24–27) and tumor tissue explants (28, 29). However, 3D tumor tissue explant culture seems to be more promising as it retains an intact tumor microenvironment.

In this study, we employed PDXs to develop and optimize a 3D tumor tissue explant culture and named it tumor *ex vivo* analysis (TEVA). TEVA, which is based on PDXs and 24 h of drug exposure, is reproducible, reliable, efficient, and rapid. The TEVA setting allows testing of numerous drugs and combinations in a robust manner, and predicts multiple drug responses accurately, as compared to *in vivo* treatment of the PDX. TEVA approach is different from other *ex vivo* approaches by putting emphasis on both (i) even size and volume of relatively large explants (2 × 2 × 2 mm^3^) allowing uniformity, reproducibility and a less divergent stroma/tumor ratio among tested samples; and (ii) performing the assay on tumor source taken from PDX (preference to first generation PDX), thus allowing testing numerous single drugs and combinations in a robust manner and after having the genomics data. Overall, our data provides a potential rationale to develop TEVA as a predictive assessment of tumor response to therapy in HNC.

## Materials and Methods

### Sample Procurement

Six head and neck cancer patients were included in the study. Details of the patients are given in Table 1. Fresh tumor tissue samples were procured just after their surgery with patient consent and with Helsinki approval from Ear Nose and Throat unit, Soroka Medical Center, Israel and Rabin Medical Center, Israel. The numbers of the Ethics Committee approvals are 0372-15-SOR, 0421-16-SOR, 0103-17-SOR, and 0813-16-RMC. The samples were placed in serum free DMEM (Gibco) media for transport and then processed within 2–3 h from harvesting.

**Table 1:** Summary of the patients' tumor characteristics used for this stud.

**Patient code**	**Place of tumor**	**Grade**	**Stage**
SE-Pt#1	Glottis (Larynx)	Moderately differentiated	T2N0M0
SE-Pt#2	Hypopharynx	Advanced	NA
SE-Pt#3	Tongue (Oral cavity)	Moderately differentiated	T0N2CM0
SE-Pt#4	Nostril (Skin)	Moderately differentiated	T3N0M0
SE-Pt#5	Glottis (Larynx)	Moderately differentiated	T2N1M0
SE-Pt#6	Glottis (Larynx)	Moderate-poorly differentiated	T3N20M0

### Mice and Generation of PDXs

Male NOD/SCID (Envigo-NOD.CB17-Prkdcscid/NCrHsd) mice were used for the study. The patient-derived tumor tissue samples were implanted subcutaneously in dorsal flanks of the mice to form the PDXs. Tumor take rate varied from 1 to 6 months. PDXs were maintained by passing the tumors in mice from first generation to subsequent generations. All animal experiments were done under Institutional Animal Care and Use Committee (IACUC) of Ben-Gurion University of the Negev (BGU's IACUC) according to specified protocols aiming to ensure animal welfare and reduce suffering. The Animal ethical clearance protocol number used for this research is IL-80-12-2015.

### *Ex vivo* Tissue Explant Preparation and Culture

When the PDXs (preferably first generation PDX) reached ~500 mm^3^, they were subjected to Tumor *ex vivo* Analysis (TEVA). After excising out the PDXs aseptically from mice they were cut into 2 × 2 × 2 mm^3^ tissue explants and cultured in 48 well tissue culture plates for specific time point as indicated in the text. The DMEM culture media (Gibco) contained 20% FBS (Gibco), 1 mM sodium pyruvate (Biological Industries), 2 mM L-glutamine (Biological Industries), 1% penicillin/streptomycin/amphotericin (Biological Industries), 0.1 mM MEM non-essential amino acids (Biological Industries), 10 mM HEPES (Biological Industries), 1% BIO-MYC (Biological Industries) and 50 ug/ml gentamycin (Gibco). For drug treatment, the 2 × 2 × 2 mm^3^ explants were treated with different therapeutic drugs for 24 h in 48 well tissue culture plates in 37°C, 5% CO_2_.

### Therapeutic Agents

BYL719 (2.5 uM) (AdooQ bioscience, A11328), Erlotinib (5 uM) (MedChem Express, HY-50896), Cetuximab (10 ug/ml) (Merck, kindly provided by Soroka Medical Center), Cisplatin (9 uM) (Teva Pharmaceutical Industries), Olaparib (5 uM) (MedChem Express, HY-10162), and 5FU (123 uM) (Sigma, F6627) were used for the study.

### Tissue Microarray (TMA)

The treated or untreated 2 × 2 × 2 mm^3^ tumor tissue explants were then went through formalin fixed paraffin embedding (FFPE) process using automated tissue processing machine (Leica, Biosystems) as we previously described (30, 31). TMA blocks containing up to 24 tissues were made from donor paraffin tissue blocks using 3 mm T-Sue™ punch needles (Simport).

### Immunohistochemistry Staining and Quantification

5 μm sections were cut from TMA blocks using fully automated rotary microtome (Leica RM2255) and then Hematoxylin (Mayer) and Eosin (1% alcoholic) [Pioneer research chemical Ltd, UK] staining was performed as described (32). For (IHC) staining, tissue sections were first deparaffinized by two rinses in xylene and 100% ethanol for 10 min each. After subsequent rinses in 70 and 50% ethanol for 5 min tissues were washed in ultrapure water and subjected to antigen retrieval at 95°C for 30 min using antigen unmasking solution, citrate buffer P.H 6.0 (Invitrogen). Sections were then washed and ImmPRESS universal reagent (Vector Laboratories, MP-7500) was used according to manufacturer's protocol for the blocking. After the blocking, sections were incubated with primary antibodies against human cytokeratin 14 (1:2,000, Abcam, cat no-ab181595), vimentin (1:100, Cell signaling Technology, cat no-5741), α-SMA (1:250, Abcam, cat no-ab5694), Ki67 (1:250, Vector laboratories, cat no-VP K451), p-ERK (1:250, Cell signaling Technology, cat no-4370) p-PRAS40 (1:250, Cell signaling Technology, cat no-2997). For the HRP conjugated second antibody step we followed the manufacturer protocol of ImmPRESS universal reagent. Then immunoreactivity was visualized by using DAB substrate kit (Cell Marque, cat no-957D-60), according to manufacturer protocol. TUNEL assay was done according to manufacturer protocol (TREVIGEN, cat no-4815-30-K). TMA images were taken by pannoramic scanner (3D Histech). Images were analyzed by HistoQuant™ software (3D Histech). For the analysis, tumor areas were annotated based upon cytokeratin 14 staining of images of the respective PDXs. For both Ki67 and TUNEL, the software can calculate the number of positive nuclei and the annotated area for each tissue and the value was expressed as object frequency (pcs/mm^3^). For p-ERK, p-S6, and p-PRAS40, the software calculated proportion of the stained area within the annotated area for each tissue, and the value were expressed as relative mask area (%).

### Western Blotting

2 × 2 × 2 mm^3^ tumor tissue explants were lysed group wise with 50 mM HEPES (pH 7.4), 100 mMNaCl, 0.1% CHAPS, 1 mM DTT, and 0.1 mM EDTA and protease inhibitor cocktail (Calbiochem) containing buffer to extract the total cellular protein. Protein concentration was determined by Bradford assay (BIO-RAD, Protein Assay, cat no-500-0006). 30 μg of total cellular protein from each group were electrophoresed on 10% SDS–polyacrylamide gels followed by their transfer onto nitrocellulose membranes. The membranes were blocked with 10% skimmed milk in Tris Buffered Saline with Tween 20 (TBST) for 2 h at room temperature, followed by overnight incubation at 4°C with p-AKT (Cell signaling Technology, cat no-4060), p-ERK and β-actin (MP Biomedicals, cat no-0869100) diluted 1:1,000 in 1% BSA in TBST. Blots are then washed with TBST followed by incubation with Goat Anti-Rabbit IgG Polyclonal Antibody (HRP) (KPL) and Peroxidase AffiniPure Rabbit Anti-Mouse IgG (H + L) (*Jackson ImmunoResearch* LABORATORIES, INC.) at a dilution of 1:5,000 in TBST. SuperSignal West Pico Chemiluminescent Substrate (Thermo Scientific™) was used for developing blots and the images were taken using XRS+ imaging system (Bio-Rad). Densitometry analysis of the blots was done using ImageJ software. Values of p-AKT and p-ERK were represented normalized to β-actin (33).

### *In vivo* Drug Efficacy

Patient derived xenografts were re-implanted subcutaneously into mice and when the tumors reached ~100 mm^3^, mice were randomized keeping two restrictions: average PDX size and SD in the different groups should be similar, and SD should be kept lower thus excluding from the experiment, prior to its beginning, mice bearing PDX with volumes at the edges of the PDX volume range. The mice were treated as follows: BYL719 (25 mg/kg, daily, orally) (AdooQ bioscience, A11328), erlotinib (50 mg/kg, daily, orally) (Glentham Life Sciences, GP3306), cetuximab (10 mg/kg, once every 5 days, ip) (Merck, kindly provided by Soroka Medical Center), cisplatin (2 mg/kg, twice weekly, ip) (Teva Pharmaceutical Industries), olaparib (50 mg/kg, daily, orally) (Glentham Life Sciences, GP0126), and 5FU (8 mg/kg, thrice weekly, ip) (Sigma, F6627). Tumor volume was measured on day 1 and thereafter every 3 days using digital calipers. Formula used for tumor volume calculation is (L × W × W) × (π/6) in which W is the smaller measurement of tumor size.

### *In vivo* and *ex vivo* Scoring

Calculation of VitroF was based on the staining of Ki67 and TUNEL of tissue treated with control- DMSO (Ct) and treatment (Tr),

$$\begin{array}{l} \text{ TEVA score }(\text{VitroF})=(0.5\times \text{C}{\text{t}}_{\text{Ki}67}/\text{T}{\text{r}}_{\text{Ki}67})\\ \;+\;(0.5\times \text{T}{\text{r}}_{\text{TUNEL}}/\text{C}{\text{t}}_{\text{TUNEL}}).\\ \text{ C}{\text{t}}_{\text{Ki}67}(\text{Control Ki}67)=100\\ \text{T}{\text{r}}_{\text{Ki}67}(\text{Treatment Ki}67)=\text{Normalized value respect to control}. \end{array}$$

Calculation of VivoF was based on the rate of tumor growth on day 6 and end day of the experiment: Control Tumor Volume (CTV) and Treatment Tumor Volume (TTV).

$$\begin{array}{l} \text{ Invivo score }(\text{Vivo F})=(0.5\times \text{CTV}/\text{TT}{\text{V}}_{2\text{nd last measurement}})\\ \;+\;(0.5\times \text{CTV}/\text{TT}{\text{V}}_{\text{lastmeasurement}}).\\ \text{ CTV}(\text{Control Tumor Volume})=100\\ \text{TTV}(\text{Treatment Tumor Volume})=\text{Normalized value respect to control}. \end{array}$$

### Statistical Analysis

Graphical and statistical analyses were performed using GraphPad Prism 5.0 software. Statistical analysis of the data was performed using *t*-test, one way and two way ANOVA and including Bonferroni multiple comparison test as a *post-hoc* analysis after the ANOVA (with *p*-values of ^*^ < 0.05, ^**^ < 0.01 or ^***^ < 0.001 as indicated on the figures).

## Results

### Optimization of Culture Timings For TEVA

**Figure 1:**
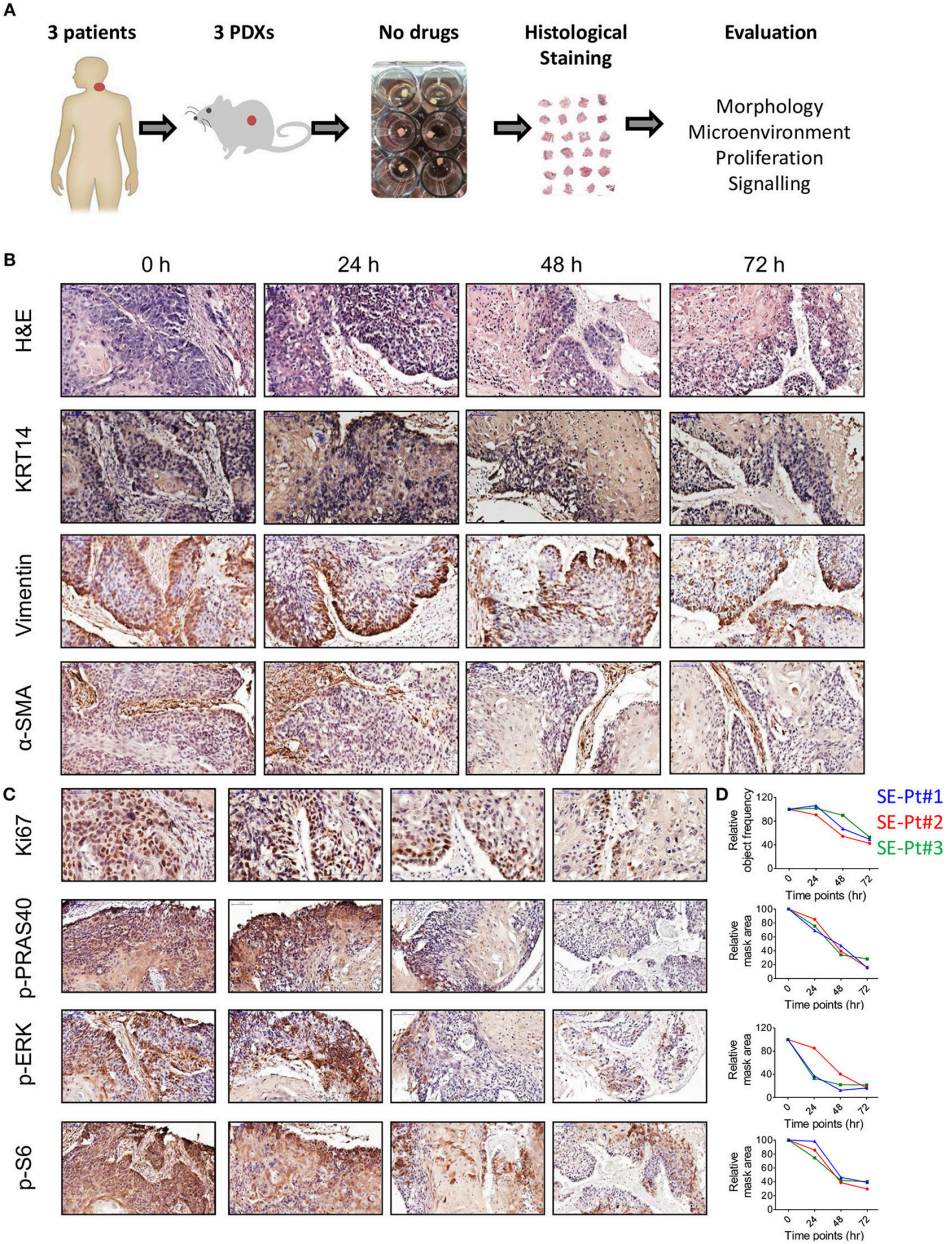
*Ex vivo* 2 × 2 × 2 mm^3^ tissue explants retain tissue morphology, tissue microenvironment, cell proliferation and cell signaling pathways. **(A)** Schematic diagram of the work flow. 2 × 2 × 2 mm^3^ tissue explants cut from the xenograft tumors of 3 HNC patients (SE-Pt#1, SE-Pt#2, and SE-Pt#3) were cultured for 24, 48, and 72 h in 400 μl DMEM media. At 0, 24, 48, and 72 h, explants were fixed, embedded and stained. **(B)** 2 × 2 × 2 mm^3^ tissue explants retain tumor morphology and tumor microenvironment. The tissue explants were stained for hematoxylin and eosin (H&E) and IHC staining was done for epithelial tumor cell marker, cytokeratin 14, fibroblast cell marker, vimentin and α-SMA (Magnification = 20×, bars = 100 μm). **(C)** 2 × 2 × 2 mm^3^ tissue explants retain cell proliferation and cell signaling pathways. The tissue explants were stained for cell proliferation marker Ki67 and cell signaling molecules p-PRAS40, p-ERK and p-S6 (For p-PRAS40, p-ERK and p-S6, magnification = 20×, bars = 100 μm and for Ki67, magnification = 40×, bars = 50 μm). **(D)** Graphs showing quantification of Ki67 positive cells; p-PRAS40, p-ERK and p-S6 positive areas in all three patient xenograft tumors at 0, 24, 48, and 72 h, respectively. Images were captured by pannoramic scanner and analysis was done by HistoQuant™ software.

To identify the appropriate culture timings for TEVA, we studied tumor tissue from PDXs of 3 patients; SE-Pt#1, SE-Pt#2, and SE-Pt#3. After cutting the PDXs to 2 × 2 × 2 mm^3^ explants, they were cultured till 72 h in DMEM with 20% FBS culture media, and pathological analysis was performed. The work flow is shown schematically (Figure 1A). The tumor tissue structure of the explants was examined by hematoxylin and eosin (H&E) staining and tumor marker of cytokeratin 14 expression. Representative images from SE-Pt#2 showed that the tissue structure is well preserved after 24 h of culture, while longer culture for 48 and 72 h resulted in tissue degradation (Figure 1B, first and second panel lines). Further analysis showed no significant changes in the tumor microenvironment after 24 h of culturing as the level of vimentin and α-SMA, fibroblast markers remained unaltered (Figure 1B, third and fourth panel lines).

Cell proliferation status was evaluated by Ki67 expression. Representative images from SE-Pt#2 showed no significant changes in Ki67 levels after 24 h of culture, while decrease in cell proliferation was observed after 48 and 72 h (Figure 1C, first panel line). Signaling activation of two key survival pathways was checked; the AKT/mTOR and MAPK. Quantification of p-PRAS40/p-S6 levels (indicators for AKT and mTOR, respectively), and of p-ERK (indicator for MAPK pathway), confirmed that after 24 h of culturing tumor tissue remain viable and active. To reinforce our observation, we validated our results in two other independent PDXs (SE-Pt#1 and SE-Pt#3) (Figure 1D). Overall the results showed that within the first 24 h of culture, cells in the tissue explants were able to maintain their viability, which allow us to conduct drug test.

### Signaling Pathways Can Be Targeted in TEVA

**Figure 2:**
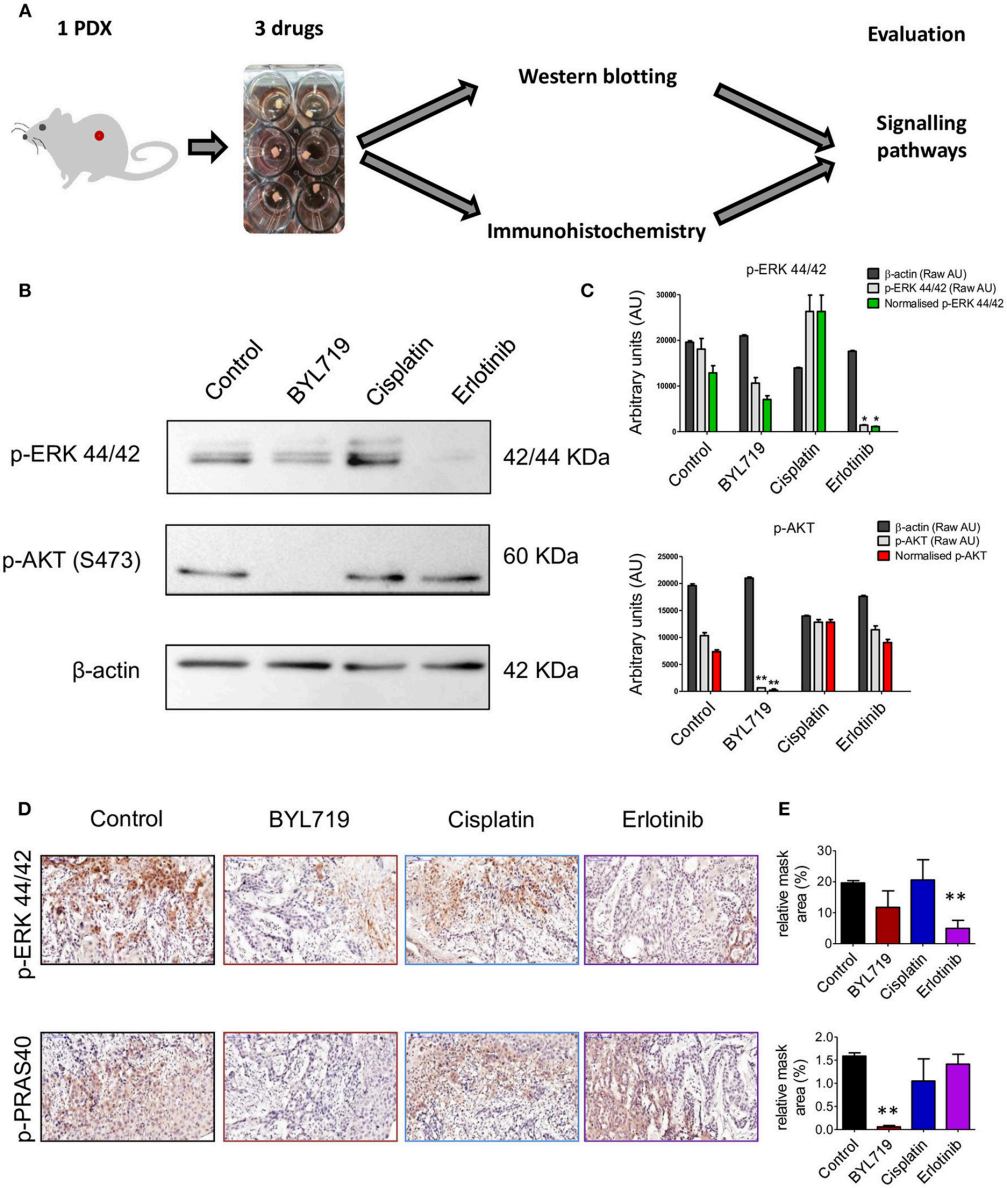
Signaling pathways can be targeted in *ex vivo* 2 × 2 × 2 mm^3^ tissue explants culture. **(A)** Schematic diagram of the workflow. 2 × 2 × 2 mm^3^ tissue explants from the xenograft tumor of SE-Pt#1 were cultured with EGFR inhibitor, erlotinib, PI3Kα inhibitor, BYL719 and chemotherapeutic drug, cisplatin for 24 h. Western blotting and immunohistochemistry were done to check the expression levels of the signaling proteins. **(B)** Western blotting was done with the protein lysates extracted from the tissue explants with protein lysis buffer to check the expression levels of p-ERK 44/42 and p-AKT. Representative images of the immunoblots were shown. **(C)** Representative graphs of densitometry analysis of the depicted immunoblots. Results are shown as arbitrary units (AU) of control β-actin, p-AKT, and p-ERK 44/42, obtained by densitometry analysis; Results are given both in raw AU (gray-shaded columns) and in β-actin-normalized AU (red/green color-shaded columns). **(D)** IHC staining was done from FFPE tissue explants to check the expression levels of p-ERK and p-PRAS40. Representative images were shown (magnification = 20×, bars = 100 μm). **(E)** Representative graphs showing quantification of p-ERK and p-PRAS40 positive areas of IHC images. Graphs representing mean ± SEM. *p < 0.05, **p < 0.01, Student's *t*-test, significantly different from control.

To investigate if tumor tissue explants from PDXs can be used for *ex vivo* drug efficacy, effects of chemotherapy and two targeted therapies on signaling pathway inhibition was performed on SE-Pt#1. The 2 × 2 × 2 mm^3^ explants were cultured with a chemotherapeutic agent, cisplatin, an EGFR inhibitor, erlotinib, and a PI3K inhibitor, BYL719 for 24 h followed up by western blot and pathological analysis (Figure 2A). Western blotting analysis indicates that erlotinib inhibited MAPK pathway, indicated by reduction of p-ERK, while BYL719 inhibited the AKT pathway shown by reduction of the phosphorylation of AKT. IHC analysis of the tumors confirmed that the drug penetrated into the tumor, as the signaling pathways of tumors were inhibited with these agents. As expected, cisplatin exhibited no effect on AKT and MAPK pathways as the levels of p-AKT/p-PRAS40 and p-ERK levels sustained (Figure 2B). These pathway inhibition results were significantly evidenced by image analysis of the western blot and IHC results (Figures 2C–E, respectively).

### Correlation of Drug Testing Results From *ex vivo* and *in vivo* Assays

**Figure 3:**
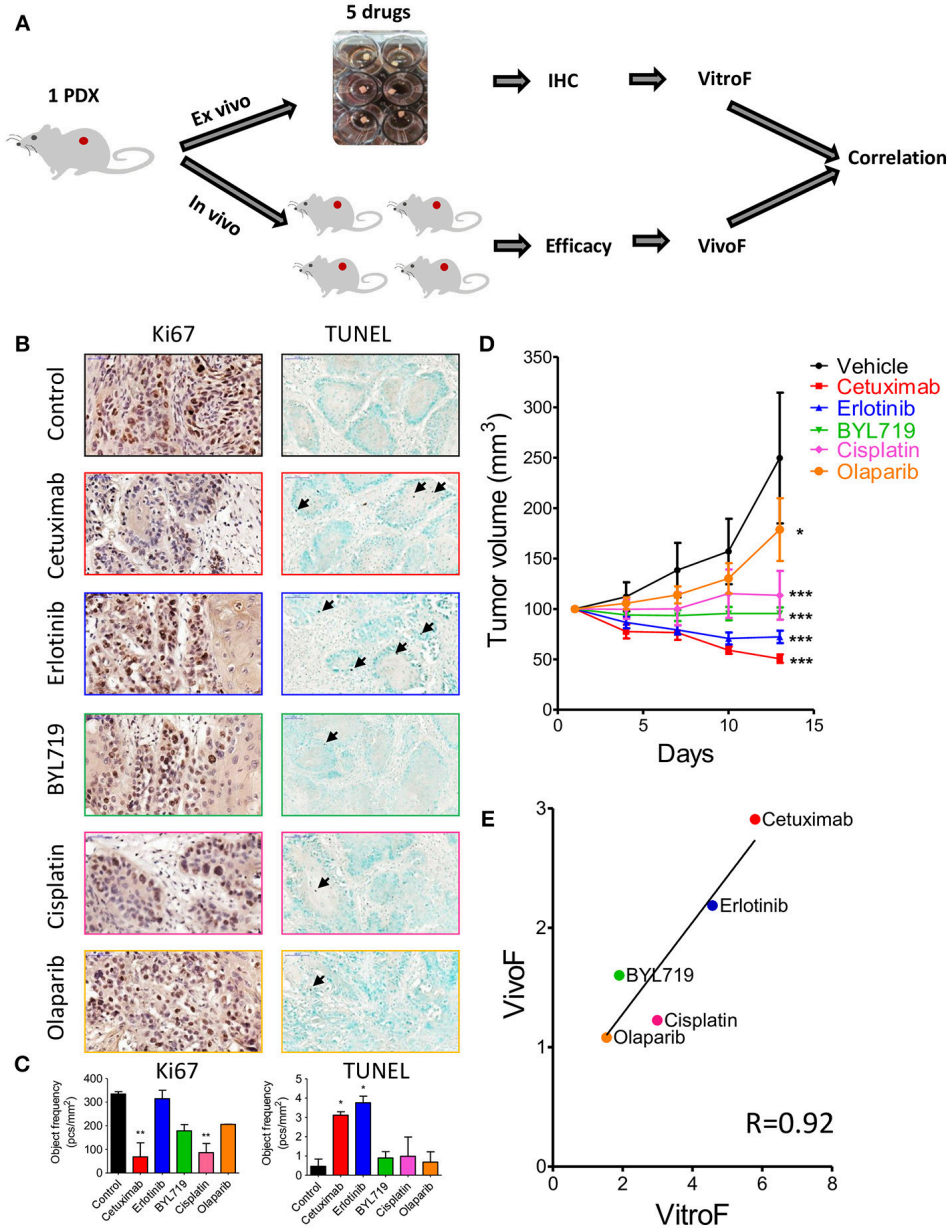
Response of 2 × 2 × 2 mm^3^ tissue explants of SE-Pt#4 to multiple therapeutic drugs correlates with the *in vivo* results. **(A)** Schematic diagram of the work flow. 2 × 2 × 2 mm^3^ tissue explants from the xenograft tumor of SE-Pt#4 were treated with cetuximab, erlotinib, BYL719, cisplatin, and olaparib for 24 h. TEVA score (VitroF) was derived based on IHC staining and analysis of Ki67 and TUNEL. *In vivo* score (VivoF) was derived based on efficacy studies on the xenograft bearing mice. **(B)** Representative IHC images of control and treated explants showing cell proliferation marker, Ki67 (magnification = 40×, bars = 50 μm) and apoptotic marker, TUNEL (magnification = 20×, bars = 100 μm). **(C)** Graphs showing quantification of Ki67 and TUNEL positive cells of control and treated explants, expressed as object frequency (pcs/mm^2^). Graphs representing mean ± SEM. *p < 0.05, **p < 0.01, One-way ANOVA with Bonferroni's *post-hoc* test, significantly different from control. **(D)** SE-Pt#4 xenografts bearing NOD/SCID mice were treated with vehicle, cetuximab, erlotinib, BYL719, cisplatin, and olaparib for 13 days. Tumor volumes (mm^3^) of vehicle and drug treated mice were shown in the tumor growth curve. Graphs representing mean ± SEM. *p < 0.05, ***p < 0.001, Two-way ANOVA with Bonferroni's *post-hoc* test, significantly different from vehicle. **(E)** Correlation data between VitroF and VivoF for each drug in SE-Pt#4 (GraphPad 5.0). The statistical significance of the correlation was determined using the correlation coefficient (*R* = 0.92). A linear regression line is shown in the plot.

After establishing our explant culture system, we sought to explore if 24 h of *ex vivo* treatment will provide an indication on the potency of therapy *in vivo*. In order to do that, we tested side by side the effect of five different therapeutic agents (cetuximab, erlotinib, BYL719, cisplatin, and olaparib) on tumor cell viability *ex vivo* and tumor volume *in vivo* (Figure 3A). For this purpose we used a PDX (SE-Pt#4). For *ex vivo* cell viability staining, IHC of Ki67 (proliferation), and TUNEL (apoptosis marker) were performed. Figure 3B showed representative images of Ki67 and TUNEL positive cells of untreated control and treatment groups. Quantification of the Ki67 and TUNEL positive cells was performed by HistoQuant™ software. Note that between different FFPE cuts of the same explant and among different explants, the ratio of tumor vs. stroma could be different. Therefore, quantitative results for Ki67 and TUNEL staining are expressed as object frequency (pcs/mm^2^), which normalizes the results only for the tumor area in the scan (see Materials and Methods). We observed that of the 5 drugs tested, cetuximab treatment *ex vivo* showed the best effect in Ki67 assay and second-best in TUNEL, while erlotinib treatment showed the best effect in TUNEL assay and the worst effect in Ki67 (Figure 3C). These differences could represent a different balance in the drugs' effectiveness on proliferation vs. cytotoxicity. Therefore, we devised a formula that take into account both assays and defined a TEVA score (VitroF, see Materials and Methods) to assess the *ex vivo* results.

To validate the drug screening results of TEVA *in vivo*, efficacy studies in NOD/SCID mice were performed. The PDX-bearing mice were allocated into 6 groups and treated with vehicle, cetuximab, erlotinib, BYL719, cisplatin, and olaparib for 13 days. Tumor volume was measured every 3 days (Figure 3D). To assess the *in vivo* effect and better compare it to the VitroF score of the *ex vivo* assay, we formulated an *in vivo* effect score termed as VivoF. Basically, the first and last measurements for each PDX were normalized to the average of vehicle-treated group and formulated together to give the VivoF (see Materials and Methods). We then compared VitroF and VivoF (Figure 3E). Cetuximab treatment was the most potent treatment as assessed by both VitroF and VivoF and regression curve showed a significant correlation between VitroF and VivoF (*R* = 0.92) for all drugs. These results of SE-Pt#4 suggest that TEVA can successfully predict multiple drug responses.

**Figure 4:**
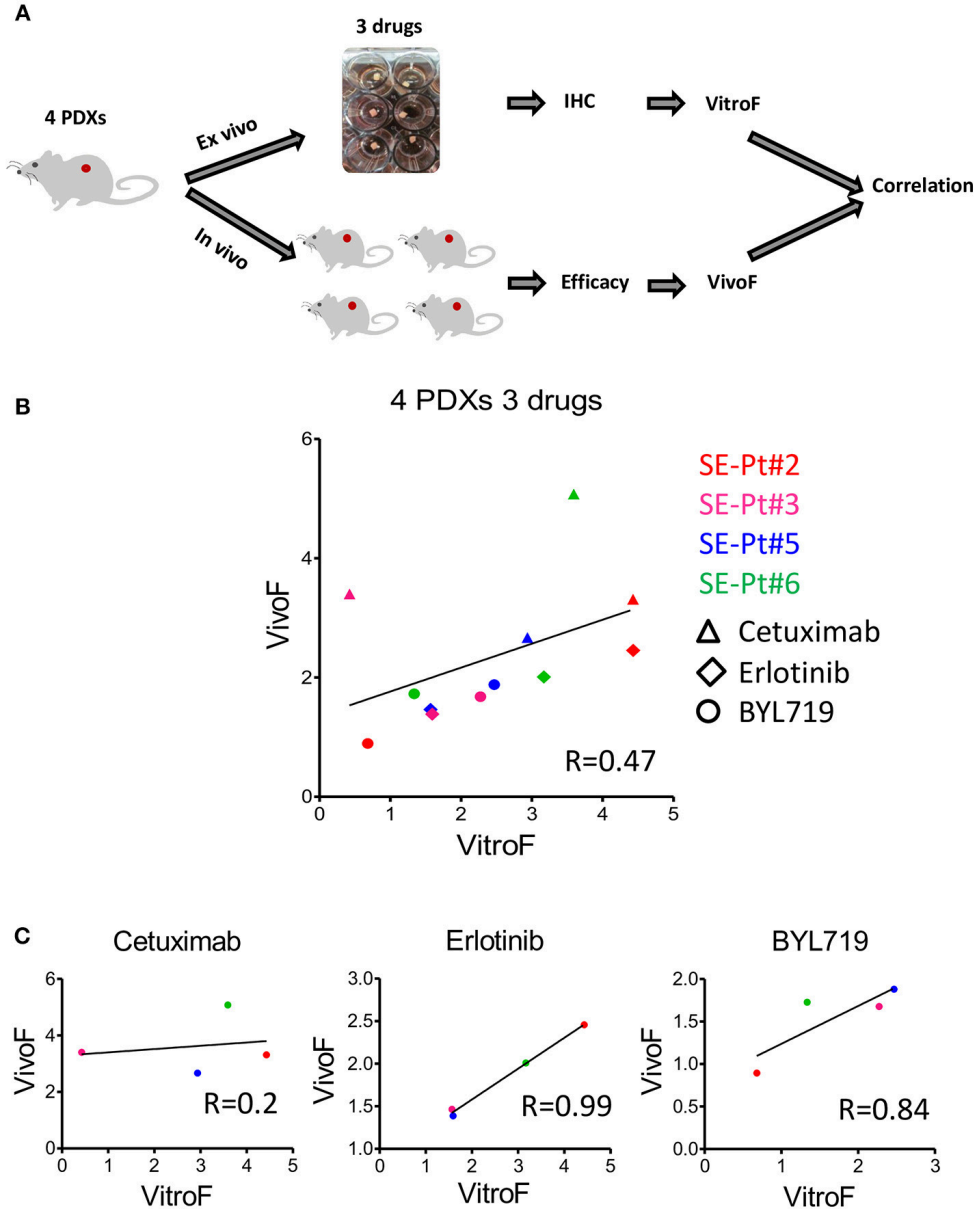
TEVA score correlates with the *in vivo* score for multiple drugs in multiple PDXs. **(A)** Schematic diagram of the work flow. 2 × 2 × 2 mm^3^ tissue explants from the xenograft tumors of four patients (SE-Pt#2, SE-Pt#3, SE-Pt#5, and SE-Pt#6) were treated with cetuximab, erlotinib, and BYL719 for 24 h. TEVA score (VitroF) was derived based on IHC staining and analysis of Ki67 and TUNEL. *In vivo* score (VivoF) was derived based on efficacy studies on the xenografts bearing mice of the four patients in different xenograft specific experiments. **(B)** Correlation data between VitroF and VivoF among three drugs in four patients (GraphPad 5.0). The statistical significance of the correlation was determined using the correlation coefficient (*R* = 0.47). A linear regression line is shown in the plot. **(C)** Correlation data between VitroF and VivoF in cetuximab (*R* = 0.2), erlotinib (*R* = 0.99), and BYL719 (*R* = 0.84) in four patients were shown (GraphPad 5.0). The statistical significance of the correlation was determined using the correlation coefficient. A linear regression line is shown in each plot.

To validate the *ex vivo–in vivo* correlation for multiple drugs observed for SE-Pt#4, we tested PDXs from 4 other patients (SE-Pt#2, SE-Pt#3, SE-Pt#5, and SE-Pt#6) with parallel treatment of 3 therapeutic drugs (Cetuximab, erlotinib, and BYL719). Figure 4A shows schematic diagram of the work flow. As described earlier, Ki67 and TUNEL were chosen to predict the drug responses in TEVA. To validate the drug screening results in TEVA, *in vivo* studies were done with respective xenografts bearing NOD/SCID mice. The mice were allocated in each experiment into four groups and treated with vehicle, cetuximab, erlotinib, and BYL719 for 15 days. Tumor volume was measured every 3 days. VitroF and VivoF were derived for each drug as described earlier. Overall the results showed a correlation between VitroF and VivoF (*R* = 0.47) for three drugs in four PDXs (Figure 4B). For erlotinib and BYL719, *ex vivo–in vivo* results are highly correlated whereas for cetuximab the level of correlation is not high. Results also showed that for erlotinib, SE-Pt#2 was most sensitive whereas SE-Pt#3 and SE-Pt#5 were less sensitive. On the other hand for BYL719, SE-Pt#5 was most sensitive whereas SE-Pt#2 was least sensitive (Figure 4C). These suggest that TEVA results are in concordance with the patient derived xenografts responses.

### Drug Combinations Can be Screened in TEVA

**Figure 5:**
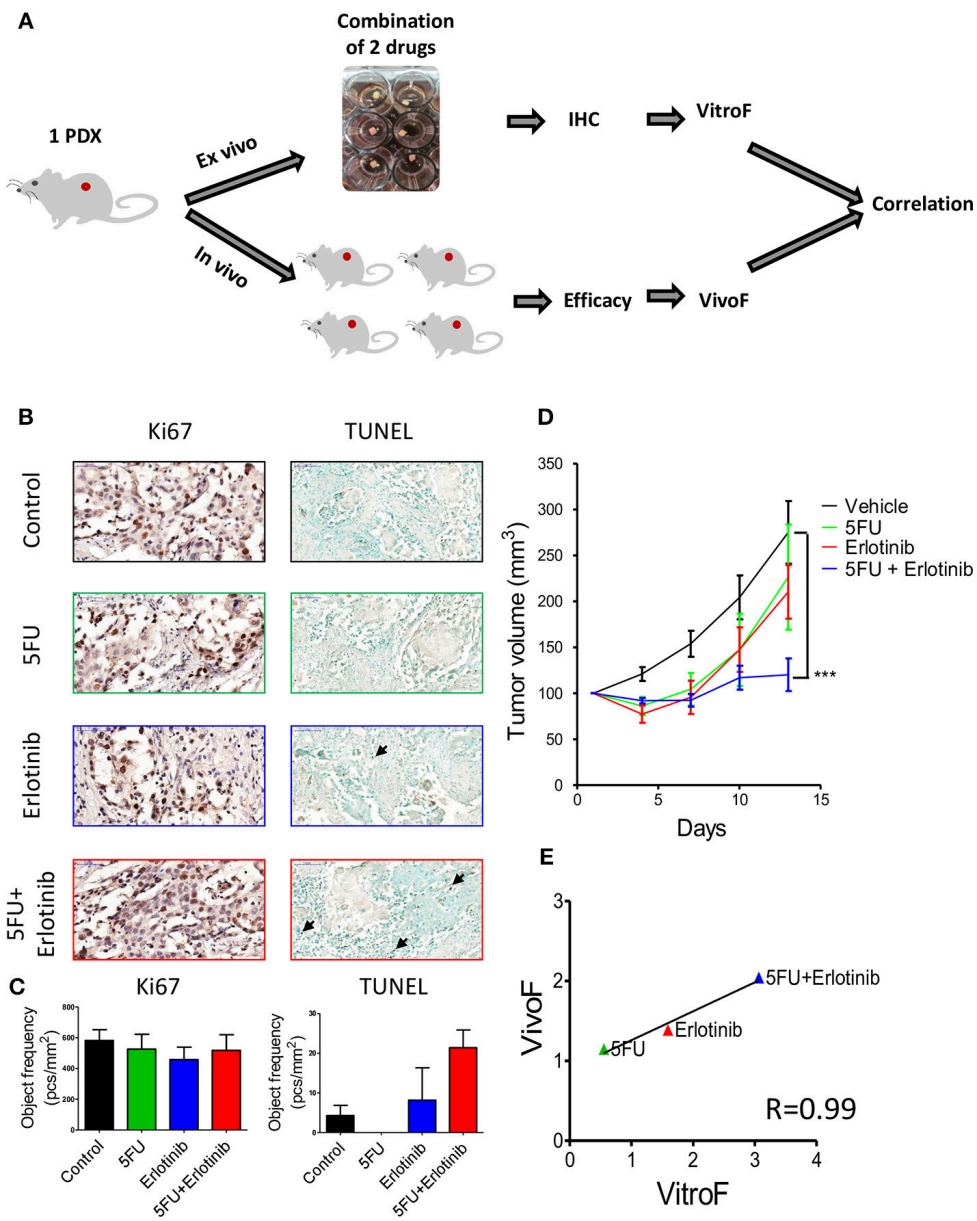
Response of 2 × 2 × 2 mm^3^ tissue explants of SE-Pt#3 to combination of two drugs correlates with the *in vivo* results. **(A)** Schematic diagram of the work flow. 2 × 2 × 2 mm^3^ tissue explants from the xenograft tumor of SE-Pt#3 were treated with 5FU, erlotinib and combination of 5FU and erlotinib for 24 h. TEVA score (VitroF) was derived based on IHC staining and analysis of Ki67 and TUNEL. *In vivo* score (VivoF) was derived based on efficacy studies on the xenograft bearing mice. **(B)** Representative IHC images of control and treated explants showing cell proliferation marker, Ki67 (magnification = 40×, bars = 50 μm) and apoptotic marker, TUNEL (magnification = 20×, bars = 100 μm). **(C)** Graphs showing quantification of Ki67 and TUNEL positive cells of control and treated explants, expressed as object frequency (pcs/mm^2^). **(D)** SE-Pt#3 xenografts bearing NOD/SCID mice were treated with vehicle, 5FU, erlotinib and combination of 5FU and erlotinib for 14 days. Tumor volumes (mm^3^) of vehicle and drug treated mice were shown in the tumor growth curve. Graphs representing mean ± SEM. ***p < 0.001, Two-way ANOVA with Bonferroni's *post-hoc* test, significantly different from vehicle. **(E)** Correlation data between VitroF and VivoF for each drug in SE-Pt#3 (GraphPad 5.0). The statistical significance of the correlation was determined using the correlation coefficient (*R* = 0.99). A linear regression line is shown in the plot.

Finally we sought to test TEVA responses to combination of targeted and chemotherapeutic drugs. For this study, we chose SE-Pt#3 that displayed a modest response to erlotinib (Figure 4). To enhance the anti-tumor activity of erlotinib, it was combined with the chemotherapeutic drug, 5FU (Figure 5A). Figure 5B showed representative images of Ki67 and TUNEL positive cells of untreated control and treatment groups. Quantification of the Ki67 and TUNEL positive cells showed that the combination of erlotinib and 5FU has significantly high number of TUNEL positive cells than the control although no significant effect on Ki67 levels (Figure 5C). To validate the drug screening results in TEVA, *in vivo* studies were done with SE-Pt#3 xenografts bearing NOD/SCID mice. The mice were allocated into four groups and treated with vehicle, erlotinib, 5FU and combination of erlotinib and 5FU for 14 days. Tumor volume was measured every 3 days and represented in a tumor growth curve where the combination of erlotinib and 5FU showed significant decrease in the tumor volume with respect to vehicle (Figure 5D). VitroF and VivoF were derived for each drug as described earlier. Regression curve showed a significant correlation between VitroF and VivoF (*R* = 0.99) for each drug in SE-Pt#3 (Figure 5E), suggesting that TEVA can successfully predict responses of drug combinations.

## Discussion

Tailoring therapy based on omics has a significant impact in medical oncology that enlarges the number of therapeutic strategies (34–36). Precision medicine using targeted therapy is subjected to two major challenges: biomarker of response (beyond genomic alteration), and acquisition of resistance [reviewed in (37)]. Taking together the recent development of cancer genomic and precision medicine, oncologists face a daily problem to select the optimal and most potent drug to each cancer patient. Hence, a functional platform that will help in decision making is urgently needed. Here, we suggest such a platform that potentially can be used to screen many drugs efficiently. We showed that a rapid and a low-cost *ex vivo* system, termed as TEVA, which is based on patient's PDX material, can mimic the efficacy of therapy assessment in mice. Since TEVA can rapidly evaluate the efficacy of numerous single drugs or combination based on material harvested from only one PDX (e.g., first generation PDX), TEVA potentially can assist in guiding personalized treatment, in relatively short time and from an extended list of omics-predicted targeted chemotherapy as well as off-label drugs.

Recently many studies has been done by employing patient derived xenografts (PDXs) from HNC, breast cancer, ovarian cancer, lung cancer to urological cancer patients for preclinical studies (5, 13–17). PDXs are successful in drawing the attention of the cancer biologists as it maintains more similarities in terms of structure, genetic profile and heterogeneity to the parental tumor than 2D monolayers (38). Despite all the ingenious advantages of PDX models in oncology research, these models also have major drawbacks to predict patient outcome alone (39). To fulfill the need of *ex vivo* drug screening to predict patient outcome, many studies have been done with patient derived tumor spheroids (20, 21), 3D organoids (22, 23), tumor tissue slices (24–27) and tumor tissue explants. These models have obviously advanced our understanding of the tumor behavior and bridge the gap between cell line/xenograft studies and actual efficacy in patients (18). Moreover, drug screening in tumor tissue explants hold the promise to maintain intact tumor microenvironment. Recently an ovarian cancer study using cryopreserved tumor tissue explants showed evidence of intact tumor microenvironment. The group successfully predicted carboplatin treatment and combination of carboplatin and 4-MU response in chemo sensitive vs. chemo resistant tissues (29). Another study of lung carcinoma using fresh tumor tissue explants from patients also showed that the tissue architecture is maintained. They showed cisplatin treatment response in the explants to be correlated with the survival of the patients and their outcome (28). However, this platform needs to be more perfected for multiple therapeutic drug screening.

In our study, we have perfected the *ex vivo* tumor explant culture platform using PDXs. In HNC, expansion of tumor mass as a PDX is required due to the limited tissue volume available from the surgery. The cultivation effect on the explants showed that the most favorable time point to see the optimal drug response in our system was 24 h. To prolong the viability of the tumor explants *ex vivo*, the culture media was supplemented with different growth factors. However, for the purpose of this study, exposure of tumors to therapeutic drugs for 24 h was found to be sufficient to detect drug efficacy. Specifically, we showed a potent signaling pathway inhibition after 24 h of treatment with anti-PI3K and EGFR drugs in all parts of the 2 × 2 × 2 mm^3^ explants.

Our data also showed that TEVA can patient-specifically predict multiple drug responses as well as drug combination effects and mimics *in vivo* results. For predicting drug response in TEVA, we used the concept of tumor microarray and generated TMA blocks capable of containing up to 24 whole explants. Panoramic scanner enabled us to scan the whole TMA images, reducing the chance of biasness of taking pictures of specific areas of interest in other microscopes and quantification of the TMA images was done by HistoQuant™ software. These all techniques enabled us to compare explant responses to different therapeutic drugs along with untreated solvent control (DMSO) on one single slide. Thus, differential drug responses can be predicted more accurately by TEVA within just few days, surpassing the need of PDXs in near future. However, it will be important to evaluate the explant response in a larger cohort of patients and it will be interesting to see the explant response to resistant drugs in the patients who failed first line therapy.

Note that for head and neck cancer resected samples, the time taken from getting the resected sample to having first generation PDX model in appropriate size, varies from 2 to 4 months and from PDX model to do TEVA to get personalized therapy data usually takes 3–4 days including analysis. This timeframe might not suffice for adjuvant therapy of head and neck cancer that usually begin 6–8 weeks after surgery. However, in the context of other malignancies, this time frame allows TEVA to be feasible in (i) guiding personalized treatment in the context of adjuvant therapy given 2–4 months after surgery (when TEVA is performed from first generation PDX), (ii) following relapse (including head and neck cancers), and as aforementioned (iii) patients with advanced tumors who failed first line therapy (including head and neck cancers).

Among all the drugs tested, we found that for cetuximab alone, TEVA results not always mimics *in vivo* results. Cetuximab is an anti-EGFR human-mouse chimeric IgG1 antibody. The Fc region of cetuximab can bind to the Fc-receptors on different immune cells, including NK cells and thus to induce antibody-dependent cell-mediated cytotoxicity (ADCC) (40). Thus, cetuximab can enhance the ADCC of NK cells and studies proved that NOD/SCID mice do have 25.2% NK cells (41). This could be a possible reason of the enhanced activity of cetuximab *in vivo* irrespective of its direct effect on the tumor cells. Therefore, for chemotherapy and small molecules-based targeted therapy, we expect TEVA to fully mimic *in vivo* results. For evaluating mAb-based targeted therapy in PDX-mice vs. TEVA, we should be somewhat cautious. Human IgG1, and to a lesser extent human IgG4, can bind effectively to murine CD64 (FcγRI) (42), expressed by murine macrophages and neutrophils. Thus, we should bear in mind that for some humanized mAbs, assessed for potency of targeted therapy in PDX mice vs. TEVA, and recognizing moieties on the cancer cells, ADCC could be more efficient in the PDX-bearing mice as compared to the TEVA.

In conclusion, we developed and optimized a rapid, low-cost, reliable and reproducible PDX-based tumor *ex vivo* culture system (TEVA) which can predict multiple drug responses. It can also be used for large scale drug screening as well as a platform for preclinical evaluation of new anti-cancer drugs. Based on the availability and size, this method can be done also directly on patient samples, allowing much lesser time in predicting drug response. Thus, in future this method can be used in conjunction with genomics data or independently as a personalized system to predict patient specific drug responses.

## Author Contributions

AP and ME were behind the concept of the study. SG, AP, and ME did the study design. Data acquisition was done by SG, KK, MP, JZ, LC, KY, B-ZJ, BL, OD, AB-D, and AM. Data analysis and interpretation was done by SG, KK, LC, VL, ME, and AP. Manuscript preparation and editing was done by SG, KK, ME, and AP. Manuscript review was done by ME and AP.

### Conflict of Interest Statement

The authors declare that the research was conducted in the absence of any commercial or financial relationships that could be construed as a potential conflict of interest.
